# The Global Outbreak of *M. chimaera* Infection Following Cardiac Surgery: Another Piece of the Puzzle

**DOI:** 10.3390/pathogens14100964

**Published:** 2025-09-24

**Authors:** Savina Ditommaso, Gabriele Memoli, Francesca Anselmi, Ivan Molineris, Carla Maria Zotti, Monica Giacomuzzi

**Affiliations:** 1 Department of Public Health and Paediatrics, University of Turin, 10124 Torino, TO, Italy; 2 Department of Life Sciences and Systems Biology, University of Turin, 10124 Torino, TO, Italy

**Keywords:** heater–cooler, non-tuberculous mycobacteria, *Mycobacterium chimaera*, whole-genome sequencing, cardiac surgery

## Abstract

Invasive cases of *Mycobacterium chimaera* have been found in Europe, and beyond, and have been associated with the use of heater–cooler units necessary to regulate the temperature of blood in extracorporeal circulation during cardiac surgery, mostly due to contamination of patients by aerosol coming from the water in the tanks of the devices. An outbreak of five cases of *M. chimaera* infection associated with heater–cooler units Stockert 3T (LivaNova) was also identified in the Piedmont region (Italy). *M. chimaera* strains isolated from 10 Stockert 3T heater–cooler units used in 3 regional cardiac surgery operating rooms were subjected to whole-genome sequencing. The results were analysed according to van Ingen’s criteria. *M. chimaera* was isolated from 59% of heater–cooler units Stockert 3T (LivaNova) monitored. By whole-genome sequencing analysis performed on *M. chimaera* strains obtained from 10 heater–cooler units, four strains were classified as subgroup 1.1 or 1.8, which are the two subgroups associated with the HCU-related outbreak worldwide. Our data and their comparisons with global heater–cooler unit isolates provide further evidence of the hypothesis that contamination occurs at the production site of LivaNova following exposure to *M. chimaera*-contaminated factory plumbing systems.

## 1. Introduction

Non-tuberculous mycobacteria (NTMs) are widely present in water [1,2,3], soil [3], dust, and other natural environments. Potting soil, hospital water, and even shower heads can be rich in NTMs that can form difficult-to-eliminate biofilms [4]. Potable water in residential buildings and healthcare settings is a common source of exposure to NTMs. Contaminated water can spread through decorative fountains and water features, hydrotherapy equipment such as jetted therapy baths, ice machines, intravenous infusions or intramuscular or intradermal injections, medical equipment such as respiratory machines and bronchoscopes, as well as heater–cooler devices, shower heads, and sink faucets [5].

Large healthcare facilities present particular challenges due to their complex water systems, which can lead to water stagnation and the proliferation of microorganisms [6], including NTMs [2,7].

Human exposure to NTMs is common, but outbreaks of NTM infections are fairly infrequent, suggesting that NTMs have low-to-moderate pathogenicity and that host risk factors play an integral role in susceptibility to NTM disease [8]. Individuals with immunodeficiencies or underlying conditions are susceptible to NTM infections. NTM outbreaks typically occur in healthcare settings when procedures expose patients to contaminated water. Pulmonary infections are the most common clinical manifestation of NTM disease. Extrapulmonary infection surveillance can help detect healthcare-associated infections (HAIs) and outbreaks more quickly [5].

*Mycobacterium chimaera*, first identified in 2004, is an emerging NTM causing both pulmonary and extrapulmonary infections. Invasive *M. chimaera* infections have been reported in Europe [9,10,11,12,13], and beyond [14,15], and have been linked to the use of heater–cooler units (HCUs) used to regulate blood temperature during extracorporeal circulation in cardiac surgery, mostly due to contamination of patients by aerosol coming from the water in the tanks of the devices [10,12]. The first identified infection case linked to these devices dates back to 2012 [10,16], although through retrospective investigations, it was possible to also recognize cases that occurred previously [9,17]. The incubation period after exposure to *M. chimaera* is long, with a median of 17 months (range 3–72 months). Signs and symptoms include fatigue, fever, weight loss, prosthetic valve endocarditis, prosthetic valve infection, sternotomy wound infection, mediastinitis, and manifestations of disseminated infection (sepsis)—including embolic and immunological manifestations (e.g., splenomegaly, arthritis, osteomyelitis, spondylodiscitis, bone marrow involvement with cytopenia, chorioretinitis, pulmonary involvement, hepatitis, nephritis, myocarditis, central nervous system manifestations, and septic abscesses) [18]. There is no established therapy, and the mortality rate is approximately 50%.

The extent of the global epidemic is currently not known with precision. In order to elucidate the origin of the HCU colonization and understand the transmission dynamics of *M. chimaera* within clinical and environmental settings, van Ingen and coworkers [19] performed a comprehensive phylogenetic analysis using whole-genome sequencing (WGS) on 250 *M. chimaera* isolates collected from HCUs and patients, as well as from water systems at the HCU manufacturing site. Van Ingen extracted a set of variants specific for groups of isolates defined by the phylogenetic analysis, i.e., phylogenetic single nucleotide polymorphisms (SNPs) that are characteristic of isolate groups, subgroups, or branches. He grouped isolates together based on a maximum distance to the nearest group member of 1000 base substitutions for group attribution or 10 base substitutions for subgroup attribution; for each isolate, the mean allele frequency was calculated for each group-specific set of SNP alleles, setting a threshold of at least 5% for detection of a strain from that group.

In Italy, a death in a northern region during autumn 2018 prompted a retrospective analysis to identify all subjects infected with *M. chimaera*, as indicated by the Ministry of Health. As of 10 October 2019, 36 confirmed cases and 2 possible cases of invasive *M. chimaera* infection (according to the ECDC case definition) [20] had been reported to the Ministry of Health. The ECDC case definition for HCU-related invasive *M. chimaera* infection is based on both clinical and exposure criteria [20]. Confirmed case: a patient who meets the clinical and exposure criteria and has *M. chimaera* isolated by culture and identified by Sanger sequencing in a significant biological sample. Probable case: a patient who meets the clinical and exposure criteria and has *M. chimaera* identified by direct PCR and Sanger sequencing in a significant biological sample, or a *Mycobacterium avium* complex strain isolated by culture or by direct PCR from a significant biological sample, or histopathological detection of nongaseous granuloma and foamy/swollen macrophages with the presence of acid-fast bacilli in cardiac or vascular tissue or in a sternotomy wound specimen.

In Italy, the mortality rate for *M. chimaera* remains very high, at 58.3% among confirmed cases reported up to the October 2019 date [21].

In the Piedmont region (north-west Italy), clinical and environmental surveillance activity (identification of contaminated devices) indicated by the Ministry of Health led to retrospective identification of five confirmed infection cases, one of which underwent surgery in a facility in the Lombardy region. These infections were acquired in the operating room during cardiac surgery from 2009 until 2013. The mean time between surgery and diagnosis was 7.2 years (range 5–9 years).

Since 2017, our microbiological laboratory has operated as a regional reference service for microbiological surveillance of all HCUs and Extra Corporeal Membrane Oxygenation (ECMO) machines [22] located in seven cardiac surgery facilities across the Piedmont region. Our investigations have highlighted NTMs and *M. chimaera* in a considerable number of water samples from HCUs [23,24].

Here, we report the results of whole-genome sequencing (WGS) analysis of *M. chimaera* strains isolated from Stockert 3T HCUs used in regional cardiac surgery operating rooms, according to the method described by van Ingen.

## 2. Materials and Methods

During 2020–2024, we analysed (by NTM culture) 100 water samples from 22 Stockert 3T HCUs (LivaNova, Sorin Group Deutschland GmbH, Norderstedt, Germany) located in five regional facilities. Mycobacterial cultures were performed according to our internal method [24] on 100 mL water samples using Elite agar. All *M. chimaera* confirmed colonies were subcultured on 7H11 medium for 10–14 days at 37 °C to obtain one to three loopfuls of cells for pelleting and DNA extraction, yielding approximately 50–100 μL in volume [25]. Below are the details of the laboratory procedures.

### 2.1. Sample Collection

This study collected a total of 100 water samples from five regional health facilities. Facility A and Facility C are two hospitals, located in the western and southwestern parts of the Piedmont region, respectively, with approximately 450–600 beds. Facilities B, D, and E are three private clinics, situated in the western, southwestern, and northwestern parts of the region. These clinics are highly specialized centres with around 100 beds each. The facilities serve distinct patient populations and operate independently, with no exchange of equipment or resources between them.

Water samples were collected in sterile 1 L plastic bottles containing 20 mg of sodium thiosulphate. Samples were immediately sent to the laboratory or, if not possible, stored at a controlled temperature (5 ± 3 °C for 12–15 h). All analyses were performed within 24 h of collection.

### 2.2. Culture

NTM isolation was performed using the following procedure: 100 mL of each water sample was filtered through a mixed cellulose esters filter, and the filter was directly added to a Petri dish containing NTM Elite agar (0.47% Middlebrook 7H9 base, 0.5% glycerol, 1.3% agar, 0.4% yeast extract, 0.2% glucose, 0.5% bovine serum albumin, 0.0056% oleic acid, and 0.0494% of a mix of selective antibiotics and antifungals) (bioMérieux, Marcy-l’Étoile, France). Subsequently, the NTM Elite agar plates were incubated at 30 °C in a sealed plastic bag to prevent dehydration. Starting from the seventh day, cultures were examined weekly for seven weeks. After realizing that mycobacteria from environmental samples needed a longer time to grow, we opted to extend the incubation time from 4 weeks—as advised by the manufacturer—to 8 weeks. The results from the NTM Elite agar analyses were reported as CFU/100 mL [24].

### 2.3. Identification

The NTM colonies that grew on the Elite agar plates appeared to be small to medium in size and often took several days to weeks to become visible. Most of the colonies had a dry, rough surface (as *M. chelonae* and *M. fortuitum*), although some species showed smooth and shiny colonies (as *M. avium complex* and *M. kansasii*). Many of the NTM colonies were non-pigmented (white or cream-coloured appearance, as *M. avium* and *M. marinum*), but some produced pigmented colonies—either photochromogenic (pigmented upon exposure to light, as *M. fortuitum* and *M. smegmatis*) or scotochromogenic (pigmented in darkness, as *M. gordonae*, *M. paragordonae*, and *M. kansasii*).

In particular, colonies of *M. chimaera* grew to be small to moderate in size and had a smooth, cream or beige appearance [26]. Initially, they had a shiny surface, which became rough with prolonged incubation. Colonies grew slowly and usually took weeks to develop on solid soils. Over time, they sometimes showed a slightly rounded or convex shape and had a characteristic macroscopic appearance with slightly pigmented yellow colonies.

All colonies growing on the NTM Elite agar plates were confirmed as acid-fast bacilli by Kinyoun stain and were tested as non-tuberculous mycobacteria by Real-Time PCR (Anyplex plus MTB/NTM MDR-TB Real-Time Detection, V2.0; Seegene Technologies, Inc., Seoul, Republic of Korea) [27]. Colonies were further identified as *M. chimaera* by Real-Time PCR using the On-Demand Advanced DNA Kit for *M. chimaera* Detection by Genesig (Primer Design Ltd., Southampton, UK) [23].

### 2.4. Whole-Genome Sequencing

Mycobacteria cells were collected from 7H11 medium, gently resuspended in a balanced salt solution (PAGE), and heat-killed at 80 °C for 20 min. After removal of the supernatant, the enriched cell pellet was subjected to immediate DNA extraction or stored frozen (e.g., at −20 °C) until extraction could begin.

Cell pellets were resuspended in 100 μL lysis buffer (15% sucrose, 0.05 M Tris-Cl, pH 8.0, 0.05 M EDTA, pH 8.0) and briefly vortexed. A quantity of 25 μL of lysozyme was added (100 mg/mL in lysis buffer, and samples were incubated for 2 h at 37 °C under gentle agitation (500 rpm). Following the lysozyme step, 3.1 μL of 20 mg/mL proteinase K and 100 μL of 10% SDS were added. The samples were then incubated for 10 min at 65 °C and subsequently stored frozen. [25].

Genomic DNA was purified from bacterial lysate by phenol/chloroform extraction followed by ethanol precipitation. A quantity of 100 ng of DNA was fragmented on the Bioruptor Pico instrument (Diagenode SA, Ougrée, Belgium) using the following settings: 30 s ON/30 s OFF pulses, easy mode, for 3 cycles. Sonicated DNA was concentrated by ethanol precipitation.

Libraries were prepared from fragmented DNA using the Watchmaker DNA Library Prep Kit (Watchmaker Genomics, Boulder, CO, USA) following the manufacturer’s instructions and sequenced on the Illumina MiSeq platform generating 2 × 150 paired-end reads.

Raw reads were quality controlled and trimmed with fastp (version 0.23.4). Variants were called with snippy (version 4.6.0, https://github.com/tseemann/snippy (accessed on 21 March 2025) against the reference genome of *M. chimaera* strain DSM 44623 (GenBank accession number CP015278.1) and then compared with *M. chimaera* group-specific SNP alleles defined by van Ingen et al. [19] (Supplementary Table S2).

[https://ars.els-cdn.com/content/image/1-s2.0-S1473309917303249-mmc1.pdf (accessed on 21 March 2025)] using bcftools (version 1.20) [28].

Sustainable data analysis was performed using Snakemake [29]. The group and subgroup classification of strains was carried out according to the method described by van Ingen and colleagues [19].

## 3. Results

**Figure 1:**
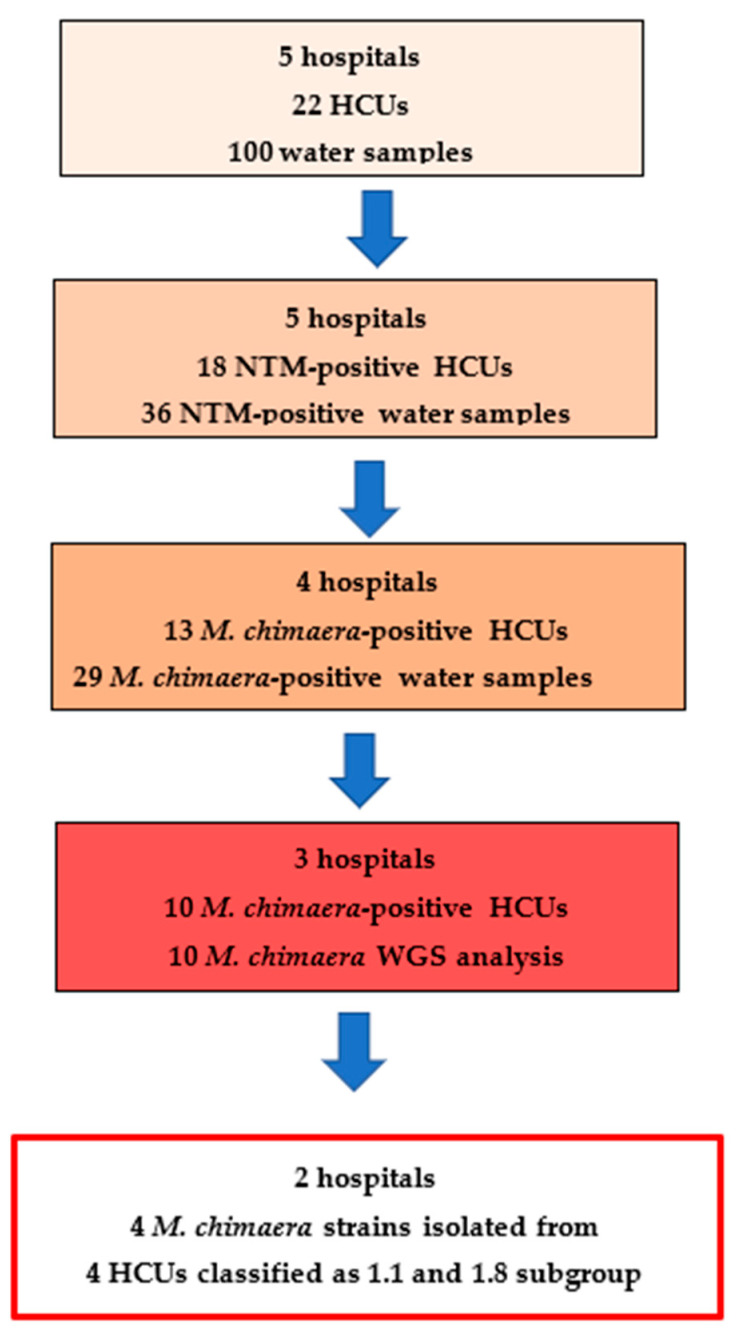
Prevalence of *M. chimaera*-positive devices in the Piedmont region and classification of sequenced strains according to van Ingen’s criteria: five years of HCU surveillance data.

NTMs were found in 36 water samples from 18 HCUs and *M. chimaera* was found in 13/22 (59%) HCUs, representing four cardiac surgeries in our region (Figure 1).

Overall, among the analysed isolates, four could be classified as 1.1 or 1.8, which are the two subgroups associated with the worldwide HCU-related outbreak. These four strains were isolated from four HCUs operating in two regional cardiac surgeries in which four patients underwent valve replacement surgery (between the years 2009 and 2013) and developed *M. chimaera* infections.

Moreover, in our study: one strain belonged to group 1 branch 2, and three strains belonged to group 2 (Table 1).

## 4. Discussion

Invasive *M. chimaera* infections have been reported in Europe [8,9,10,11], and beyond, and have been linked to the use of heater–cooler units (HCUs) mostly due to contamination of patients by aerosol coming from the water in the tanks of the devices [9,11].

Van Ingen and coworkers [21] conducted a phylogenetic analysis based on whole-genome sequencing (WGS) of 250 *M. chimaera* isolates from HCUs and patients, as well as from water systems at the HCU manufacturing site in order to understand the origin of the HCU colonization. This analysis revealed two major *M. chimaera* groups, with isolates related to cardiac surgery patients mainly classified into subgroup 1.1. Additionally, the WGS analysis demonstrated a high level of genetic similarity between isolates from the HCU factory and those from surgery-related patients, supporting the hypothesis that contamination originated from a single point source during HCU production.

In this study, the *M. chimaera* that has been assigned to subgroups 1.1 and 1.8 associated with the worldwide outbreak [10,19,30,31,32,33,34] linked to Stockert 3T HCUs were detected in four HCUs used in two regional cardiac surgery operating rooms. The literature reported that subgroups 1.1 and 1.8 were isolated from the LivaNova production site and from the majority of the analysed HCUs [33].

Our data and their comparisons with global HCU-associated isolates provide further evidence for the hypothesis that contamination occurs at the LivaNova production site, following exposure to *M. chimaera*-contaminated factory plumbing systems.

We also isolated one strain belonging to group 1 branch 2 (notably, this strain had also been found in one patient in Australia) [19] and three strains belonging to group 2.

The group 2 strains were found in three HCUs; however, these strains had never been encountered in patients who underwent open-heart surgery: we hypothesize that the strains other than those highlighted worldwide are strains that have colonized the HCUs starting from the local populations of waterborne *M. chimaera* in the plumbing systems of each facility. The remaining two strains were un-grouped because they did not exhibit any distinguishing SNP markers (Table 1).

This study has some limitations: (1) we did not store or analyse *M. chimaera* strains from all 13 *M. chimaera*-positive HCUs; (2) we analysed only one strain of *M. chimaera* for each HCU monitored. Most likely, the analysis of more strains would have given us the possibility to highlight a greater number of HCUs linked to the global outbreak; (3) we were not able to link each patient to the actual HCU adopted during cardiothoracic surgery because device serial numbers were not recorded before the international alert.

To date, no cases of postoperative invasive infections linked to Maquet devices have been reported; however, our microbiological results emphasize the need for surveillance.

In fact, surveillance of the Maquet devices adopted in our regional facilities has revealed a similar problem: widespread contamination with strains of *Mycobacterium gordonae* and *Mycobacterium paragordonae* despite repeated disinfections of the water tanks [35].

In our setting, the evidence of *M. chimaera* transmission in the cardiac surgery theatre has prompted the adoption of the best prevention practices (follow the HCU manufacturer’s instructions, microbiological surveillance of HCU water supplies, and clinical surveillance of patients undergoing cardiac surgery) in all healthcare facilities.

Following our findings, some facilities, in order to ensure patient safety, decided to move the LivaNova HCUs outside of the operating room, while others replaced their LivaNova HCUs with Maquet HCU40 devices (Maquet, Getinge Group, Rastatt, Germany).

The next effort will be to evaluate whether contamination of Maquet devices originates from a common source, like the LivaNova case, or from each facility’s individual water supply.

## 5. Conclusions

The outbreak in our region spanned several years (2009–2013) and affected only four patients.

Whole-genome sequencing revealed that our outbreak is part of the global *M. chimaera* outbreak. The high degree of similarity between our *M. chimaera* strains and those isolated from LivaNova HCUs worldwide supports the hypothesis of a single common source of infection and suggests that contamination occurred during manufacturing at the production site in Germany.

## Figures and Tables

**Table 1:** Informative SNPs and classification of sequenced *M. chimaera* isolates according to group-specific SNPs defined by van Ingen.

		Sample ID									
		MC-1180	MC-830	MC-988	MC-1271B	MC-873	MC-750	MC-791	MC-829	MC-1345	MC-872
Group/ Branch	Associated reference SNP position										
Branch_1	4977262	0	0	0	0	0	0	0	0	0	0
Branch_2	2339764	0	0	0	0	0	0	0	**1**	0	0
Branch_2	5003561	0	0	0	0	0	0	0	**1**	0	0
Group_1.1	113518	0	0	0	0	0	**1**	**1**	0	0	0
Group_1.1	209278	0	0	0	0	0	**1**	**1**	0	0	0
Group_1.11	1419163	0	0	0	0	0	0	0	0	0	0
Group_1.11	3132089	0	0	0	0	0	0	0	0	0	0
Group_1.6	4050336	0	0	0	0	0	0	0	0	0	0
Group_1.6	5033374	0	0	0	0	0	0	0	0	0	0
Group_1.8 *~	281696	0	0	0	0	0	0	0	0	0	0
Group_1.8 ~	1611282	0	0	0	**1**	**1**	0	0	0	0	0
Group_1.8 ~	2366314	0	0	0	**1**	**1**	0	0	0	0	0
Group_1 *~	2329494	**1**	**1**	**1**	0	0	0	0	0	0	0
Group_1 *~	3949608	**1**	**1**	**1**	0	0	0	0	0	0	0
Group_2.1	1828053	0	0	0	0	0	0	0	0	0	0
Group_2.1	3406341	0	0	0	0	0	0	0	0	0	0
Group_2 ~	3022332	**1**	**1**	**1**	0	0	0	0	0	0	0
Group_2 ~	5709901	**1**	**1**	**1**	0	0	0	0	0	0	0
** Sample Classification **		** Group 2 **	** Group 2 **	** Group 2 **	** Group 1.8 **	** Group 1.8 **	** Group 1.1 **	**Group 1.1**	**Branch 2**	**Un-** **grouped**	**Un** **grouped**

~ All marked SNPs are required for a specific group identification. * Reference allele is the one specific to the group. SNP: Single Nucleotide Polymorphism. MC: *Mycobacterium chimaera*. **1** indicates that the SNP variant is present; **0** indicates that it is absent. The full list of SNPs found in the 10 sequenced samples can be found at https://drive.google.com/file/d/1uzS3jXGIMV7_zYwdhoZZkp1QXpiH6AaZ/view?usp=sharing (accessed on 21 March 2025).

## Data Availability

The original contributions presented in this study are included in the article. Further inquiries can be directed to the corresponding author.
